# The TG/HDL-C Ratio Might Be a Surrogate for Insulin Resistance in Chinese Nonobese Women

**DOI:** 10.1155/2014/105168

**Published:** 2014-07-21

**Authors:** Jiyun He, Sen He, Kai Liu, Yong Wang, Di Shi, Xiaoping Chen

**Affiliations:** Department of Cardiovascular Medicine, West China Hospital, Sichuan University, Chengdu 610041, China

## Abstract

*Obejective*. To examine the discriminatory power of triglyceride (TG) and triglyceride to high-density lipoprotein cholesterol ratio (TG/HDL-C) for insulin resistance (IR) in a normoglycaemic Chinese population. *Methods*. The data were collected from 711 individuals. The normoglycaemic individuals were eventually included in the study (*n* = 533, age: 62.8 ± 6.6 years, male: 56.8%), who were with a fasting plasma glucose < 6.1 mmol/L and without a history of diabetes. IR was defined as the upper quintile (≥1.6) of homeostasis model assessment of IR. Area under the receiver operating characteristic curve (AROC) was used to examine the discriminatory power. *Results*. The discriminatory power of TG/HDL-C for IR was acceptable in women with a BMI < 24 kg/m^2^ or waist circumference < 80 cm (AROCs: 0.718 and 0.713, resp.); however, the discriminatory power was not acceptable in the obese women. TG/HDL-C was not an acceptable marker of IR in men. The discriminatory power of TG for IR was not acceptable in both men and women. *Conclusions*. The discriminatory power of TG/HDL-C for IR differs by gender and obesity index in the normoglycaemic Chinese population, and TG/HDL-C could discriminate IR in the nonobese and normoglycaemic women.

## 1. Introduction

Insulin resistance is characterized by a decrease in the ability of insulin to stimulate the use of glucose by muscles and adipose tissues and to suppress hepatic glucose production and output [1]. Insulin resistance plays an important pathogenic role in causation of diabetes and cardiovascular diseases (CVD). Therefore, identification of insulin resistance would facilitate selection of high-risk individuals for primary preventions of these interrelated diseases. Currently, the standard methods of measuring insulin resistance includes the glucose clamp, the insulin suppression test, and the frequently sampled intravenous glucose tolerance test [2–4], but these tests are not routinely measured in most clinical practices owing to the time and cost involved. Some simple methods of measuring insulin resistance includes fasting insulin, fasting plasma glucose (FPG)/fasting insulin (FINS), homeostasis model assessment method of insulin resistance (HOMA-IR), and 1/(FPG × FINS). These methods all include insulin, but plasma insulin is not routinely measured in most clinical laboratories. So, identification of insulin resistance by simple surrogates would be useful in clinical practices.

Recently, some studies [2, 5–7] gave us some hopes, and these studies showed that in patients without diabetes, triglyceride (TG) and triglyceride to high-density lipoprotein cholesterol ratio (TG/HDL-C) were closely and positively related to insulin resistance, and the two variables were recommended as surrogates for insulin resistance. However, not all studies found TG and TG/HDL-C to be associated with insulin resistance. For example, some studies [8–11] showed that TG and TG/HDL-C were not reliable markers of insulin resistance in some populations. These previous results were inconsistent, and further studies are still necessary. On the other hand, these previous studies mainly focused on non-Asian populations. Therefore, the aims of our study were to examine the discriminatory power of TG and TG/HDL-C for insulin resistance in a normoglycaemic Chinese population.

## 2. Methods

### 2.1. Study Population

In 2007, a health examination was performed in 711 individuals in an urban community located in Chengdu, Sichuan province, China. The cohort was a part of a study supported by megaprojects of science research for the 11th five-year plan, China (Trends in the incidence of metabolic syndrome and integrated control in China). The detailed information of the study has been reported elsewhere [12]. We included normoglycaemic individuals in the study, who were with a FPG < 6.1 mmol/L and without a medical history of diabetes [13]. In addition, we excluded individuals using any medication known to influence insulin resistance or lipid metabolism (such as corticosteroids and lipid-lowering drugs). Because estrogen exposure could lead to an elevation of TG levels, women receiving exogenous estrogens also were excluded. Therefore, 533 individuals with complete data (age: 62.8 ± 6.6 years, range: 45.0~83.0, male: 56.8%) were available for analysis. The study was approved by Ministry of Health of China, as well as by the Ethics Committee of West China Hospital of Sichuan University. The study protocol conforms to the ethical guidelines of the 1975 Declaration of Helsinki as reflected in a priori approval by the institution's human research committee. All participants provided written informed consent.

### 2.2. Data Collection

In 2007, medical professionals conducted a survey of CVD risk factors according to the MONICA protocol. The survey content included standardized questionnaire, physical examination, and laboratory tests. The questionnaire included sex, age and CVD risk factors, such as smoking status, alcohol consumption levels, physical activity, and CVD family history. Physical examination included blood pressure, height, weight, waist circumference, and hip circumference. Laboratory tests included FPG, total cholesterol (TC), low-density lipoprotein cholesterol (LDL-C), HDL-C, TG, high sensitivity creactive protein (hsCRP), and insulin. Blood was drawn from the antecubital vein in the morning after a 12 h fasting. Fasting serum insulin was measured by radioimmunoassay (XH-6010, Xi'an, China). Fasting glucose, TG, and HDL-C were measured enzymatically using a MODULAR P800 Analyzer (Roche Diagnostics). These chemistries were measured at the laboratory of West China Hospital (Chengdu, China).

### 2.3. Related Definitions

Insulin resistance was defined by using HOMA-IR, which was calculated as (fasting glucose mmol/L × fasting insulin mU/L)/22.5. Insulin resistant individuals were defined as those who had the highest quintile value of the HOMA-IR (≥1.6), according to the previous studies [14, 15]. Smoking is the average cigarette consumption ≥ one/day. Alcohol intake is the average intake of alcohol ≥ 50 g/day. Those with hypertension were defined as having systolic blood pressure (SBP) ≥ 140 mmHg and/or diastolic blood pressure (DBP) ≥ 90 mmHg and/or currently taking antihypertensive medications. Body mass index (BMI) was calculated as weight (in kg)/height (in  m^2^).

### 2.4. Statistical Analysis

Data are presented as means ± standard deviation (SD) for normal variables or median + interquartile range for skewed variables. Smoking and alcohol intake were used as dummy variables. Comparisons between groups were performed by independent* t* test for normally distributed variables and by the nonparametric Mann-Whitney test for skewed variables. Interactions between categorical variables were evaluated with the Pearson *χ* ^2^ test. Area under the receiver operating characteristic curve (AROC) was used to examine the discriminatory power of TG and TG/HDL-C for insulin resistance: AROC of 0.5 = no discrimination, 0.7 ≤ AROC < 0.8 = acceptable, 0.8 ≤ AROC < 0.9 = excellent, AROC ≥ 0.9 = outstanding [16]. For statistical analysis, SPSS (version 10.0; SPSS, Chicago, IL) software was used. Statistical significance was defined as *P* < 0.05.

## 3. Results

### 3.1. Demographic Data

Demographic data are presented in Table 1. Age, waist circumference, waist circumference/hip circumference, prevalence of smoking and alcohol intake, SBP, DBP, and TC/HDL-C were significantly higher in men; however, HDL-C, TG, insulin and HOMA-IR were significantly lower. TG/HDL-C, hsCRP, FPG, LDL-C, BMI, hip circumference, and prevalence of hypertension were similar in both groups. The prevalence of insulin resistance was 20.5% in the population, and the prevalence was higher in women than in men (27.4% versus 15.2%, *P* = 0.001, Table 1).

### 3.2. Discriminatory Power of TG/HDL-C and TG for Insulin Resistance in Different Population

The correlations between TG and TG/HDL-C with HOMA-IR were not similar in different groups (*r* for TG and HOMA-IR: all individuals = 0.343, men = 0.316 and women = 0.339; TG/HDL-C and HOMA-IR: all individuals = 0.376, men = 0.360 and women = 0.401; all associations, *P* < 0.001).

For discriminating insulin resistance, TG and TG/HDL-C were not acceptable in the whole population (AROCs: 0.634 and 0.654, resp.). When applied to men or women, AROCs were also not acceptable (men: 0.627 for TG, 0.657 for TG/HDL-C; women: 0.614 for TG, 0.652 for TG/HDL-C, resp.).

**Figure 1:**
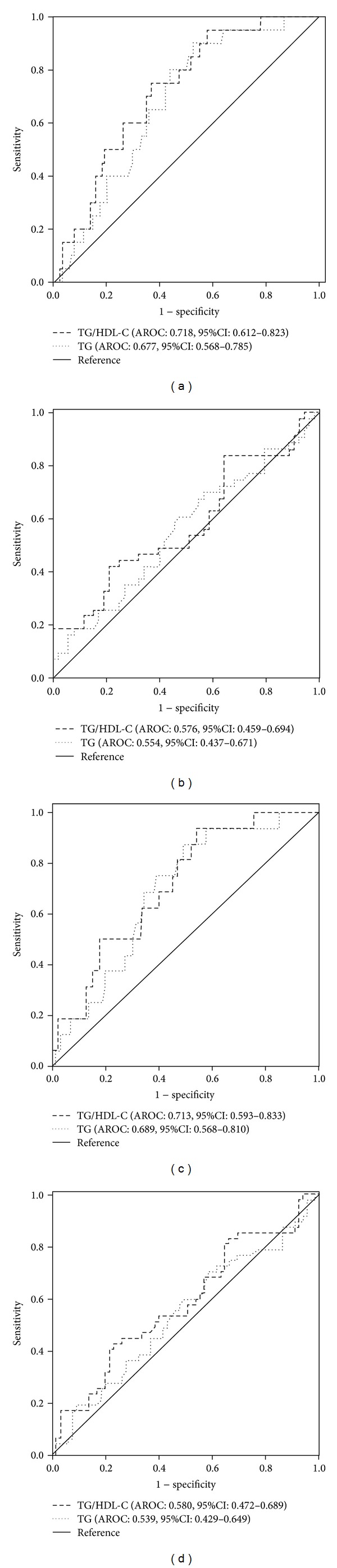
Area under the receiver operating characteristic curves of TG and TG/HDL-C for discriminating insulin resistance in women. AROCs of TG and TG/HDL-C for predicting insulin resistance in women with a BMI < 24 kg/m^2^ ((a), *n* = 134) and in women with a BMI ≥ 24 kg/m^2^ ((b), *n* = 118). AROCs of TG and TG/HDL-C for predicting insulin resistance in women with a waist circumference < 80 cm ((c), *n* = 96) and in women with a waist circumference ≥ 80 cm ((d), *n* = 112).

When women were divided into different subgroups according to BMI (<24, ≥24 kg/m^2^) or waist circumference (<80, ≥80 cm), the discriminatory power of TG/HDL-C for insulin resistance was acceptable in the nonobese women (Table 2; Figures 1(a) and 1(c)). However, the discriminatory power of TG/HDL-C for insulin resistance was not acceptable in the obese women (Table 2; Figures 1(b) and 1(d)). When men were divided into different subgroups according to BMI (<24, ≥24 kg/m^2^) or waist circumference (<90, ≥90 cm), the discriminatory power of TG/HDL-C for insulin resistance was not acceptable in each subgroup (Table 2). In addition, TG was not an acceptable marker of insulin resistance in each subgroup (Table 2, Figures 1(a)–1(d)). Further, we categorized the population into different subgroups according to the combination of BMI and waist circumference, and the discriminatory power was not improved, even lower than the single standard of classification (data not shown)

### 3.3. Other Potential Markers of Insulin Resistance

Table 3 shows AROCs for some other potential markers of insulin resistance. For men, the best surrogate for insulin resistance was waist circumference, followed by waist circumference/hip circumference, BMI, and hip circumference (Table 3). For women, BMI was the best surrogate for insulin resistance, followed by waist circumference, waist circumference/hip circumference, and hip circumference (Table 3). AROCs of all clinical variables were acceptable, and AROCs of all biological variables were not acceptable (Table 3).

## 4. Discussion

Our findings showed that the discriminatory power of TG/HDL-C for insulin resistance differs by gender and obesity index in normoglycaemic Chinese population, and the discriminatory power of TG for insulin resistance is not acceptable in both men and women. Its presence has been demonstrated in two ways. First, the discriminatory power of TG/HDL-C for insulin resistance was acceptable in the nonobese women (all AROCs > 0.700 in each subgroup). However, TG/HDL-C could not discriminate insulin resistance in the obese women, and in men (all AROCs < 0.700 in each subgroup). Second, the discriminatory power of TG for insulin resistance is not acceptable in both men and women (all AROCs < 0.700 in each subgroup).

The study showed that there were more significant associations between insulin resistance and TG, as well as TG/HDL-C, in women than in men, the same as a previous study [17]. While, Masharani et al. [18] had a different result: in women, there was no significant association between insulin resistance and TG. The current inconsistent results might be caused by sexual and racial differences in lipid profiles. Usually, women show a more favorable metabolic risk profile than men, including lower TG and higher HDL-C levels [19]. Després et al. [20] have also shown that in African Americans and white women, lipoprotein lipase (LPL) activity, which is responsible for clearing TG-containing lipoproteins from the circulation, was higher and this might induce a lower TG levels, and then further causing a weak association between TG levels and insulin resistance in those population. Along with these characteristics, some studies have shown that TG and TG/HDL-C were not reliable markers of insulin resistance in African Americans [8, 9, 11]. However, our study did not draw similar conclusions in Chinese women, and further studies should be warranted. Although sexual and racial differences might influence the associations between insulin resistance and TG, as well as TG/HDL-C, many studies have shown that increasing TG and decreasing HDL-C could deteriorate insulin sensitivity. When TG persists at high levels, heparin activates lipoprotein lipase to increase intravascular lipolysis of circulating TG, thus increasing tissue exposure to free fatty acids (FFA). High FFA may deteriorate insulin sensitivity though oxidative stress pathway [21, 22]. On the other hand, oxidation and inflammation could cause insulin resistance. Since HDL-C has the ability of anti-oxidation and anti-inflammation, decreasing HDL-C might lead to insulin resistance.

Although TG/HDL-C could discriminate insulin resistance in nonobese women, it couldn't work in obese women. A study [23] also showed that the association of TG/HDL-C with insulin resistance was stronger among people with a BMI < 25 kg/m^2^ than those with a BMI ≥ 30 kg/m^2^. Some studies [8, 10] also showed TG/HDL-C was not a reliable marker of insulin resistance in the obese. These current data might suggest that TG/HDL-C is most clinically useful for the discrimination of insulin resistance in individuals with normal weight. However, McLaughlin et al. [2] showed TG/HDL-C could discriminate insulin resistance in the subjects with a BMI ≥ 25 kg/m^2^. Current studies have different results, and confirmatory studies might be warranted. In the present study, all clinical variables were acceptable markers of insulin resistance (Table 3), and those should be recommended to be used in most clinical practices. However, all biological variables were not acceptable (Table 3).

Insulin resistance expressed by HOMA-IR is generally accepted as a valid method in epidemiological surveys. However, there is hardly any consensus on the cut-off points. Values based on 50th percentile [24, 25], 75th percentile [26], 90th percentile [27], lower boundary of the top quintile [14, 15], or tertile [2, 3] of HOMA-IR have been used previously. We defined insulin resistance as HOMA-IR greater than the 80th percentile (≥1.60), which was commonly practiced [14, 15]. In addition, when we performed a separate analyses with insulin resistance defined by the top quartile of HOMA (>1.47) or 90th percentile (>2.02), we obtained similar results (data not shown). Further, insulin resistance is reported to occur at HOMA levels that range from 2.00 to 4.00 in non-Asians, even greater [28, 29], and the threshold levels are lower in Asians, from 1.38 to 2.00 [14, 30, 31]. Our HOMA threshold of 1.6 is within this range. Although we defined insulin resistance a little arbitrarily, it might be accepted in clinical practices.

The study also had several limitations. Firstly, the major limitation of our study was failure to use a glucose clamp, an insulin suppression test, or a frequently sampled intravenous glucose tolerance test. However, those methods for all individuals over the course of our study were not feasible for pragmatic reasons and logistics. Secondly, the absence of an oral glucose tolerance test might miss some patients with diabetes. Thirdly, because of the relatively small sample size, the results of our study might have limited statistical power. No comparisons between different races might be another limitation.

## 5. Conclusion

In conclusion, the discriminatory power of TG/HDL-C for insulin resistance differs by gender and obesity index in the normoglycaemic Chinese population, and the discriminatory power of TG for insulin resistance is not acceptable in both men and women. TG/HDL-C could discriminate insulin resistance in the nonobese and normoglycaemic women, and it should be recommended in clinical practices. TG might not be recommended for clinical practices. Further studies should include different ethnic backgrounds, and the gold standard test for evaluating insulin resistance should be used.

## Figures and Tables

**Table 1:** Characteristics of the individuals.

Variables	Male (*n* = 303)	Female (*n* = 230)	*P* value
Age (years)	63.8 ± 6.2	61.5 ± 6.8	<0.001
BMI (kg/m^2^)	23.3 ± 2.9	23.5 ± 3.4	0.512
WC (cm)	83.3 ± 9.3	79.6 ± 9.9	<0.001
HC (cm)	94.8 ± 6.2	94.3 ± 6.9	0.416
WC/HC	0.9 ± 0.1	0.8 ± 0.1	<0.001
SBP (mmHg)	136.3 ± 17.9	131.7 ± 19.9	0.005
DBP (mmHg)	80.5 ± 10.1	78.0 ± 10.1	0.004
Prevalence of hypertension (%)	50.8	45.2	0.199
Smoking (%)	42.2	0.4	<0.001
Alcohol intake (%)	22.8	0.4	<0.001
TC (mmol/L)	4.7 ± 0.8	5.1 ± 0.8	<0.001
LDL-C (mmol/L)	3.0 ± 0.8	3.0 ± 0.7	0.868
HDL-C (mmol/L)	1.4 (1.2, 1.6)	1.6 ± 0.3	<0.001
TG (mmol/L)	1.4 (1.1, 2.0)	1.7 (1.2, 2.2)	<0.001
TG/HDL-C	1.0 (0.7, 1.5)	1.1 (0.8, 1.5)	0.467
TC/HDL-C	3.5 ± 0.7	3.3 ± 0.6	0.003
hsCRP (mg/L)	0.9 (0.4, 2.1)	1.0 (0.5, 2.2)	0.359
FPG (mmol/L)	4.5 ± 0.7	4.6 ± 0.6	0.218
Insulin (mU/L)	4.7 (3.4, 6.6)	5.5 (4.0, 7.9)	<0.001
HOMA-IR	1.0 (0.7, 1.4)	1.1 (0.8, 1.6)	<0.001
Prevalence of insulin resistance (%)	15.2	27.4	0.001

Data are presented as means ± SD, median (interquartile range), or percentage. BMI: body mass index; WC: waist circumference; HC: hip circumference; SBP: systolic blood pressure; DBP: diastolic blood pressure; TC: total cholesterol; LDL-C: low-density lipoprotein cholesterol; HDL-C: high-density lipoprotein cholesterol ratio; TG: triglyceride; hsCRP: high sensitivity creactive protein; FPG: fasting plasma glucose; HOMA-IR: homeostasis model assessment method of insulin resistance.

**Table 2:** Area under receiver operating characteristic curves for discriminating insulin resistance in different subgroups according to body mass index or waist circumference.

Variable	Male (*n*) (AROC, 95% CI)		Female (*n*) (AROC, 95% CI)		Male and female (*n*) (AROC, 95% CI)	
	TG	TG/HDL-C	TG	TG/HDL-C	TG	TG/HDL-C
BMI (kg/m^2^)						
<24	0.473 (*n* = 173) (0.274–0.671)	0.498 (*n* = 173) (0.293–0.703)	0.677 (*n* = 134) (0.568–0.785)	0.718 (*n* = 134) (0.612–0.823)	0.626 (*n* = 307) (0.521–0.731)	0.645 (*n* = 307) (0.542–0.749)
≥24	0.643 (*n* = 130) (0.537–0.748)	0.651 (*n* = 130) (0.549–0.752)	0.554 (*n* = 96) (0.437–0.671)	0.576 (*n* = 96) (0.459–0.694)	0.607 (*n* = 226) (0.530–0.685)	0.606 (*n* = 226) (0.528–0.684)
WC (cm)						
A Groups	0.595 (*n* = 230) (0.457–0.734)	0.632 (*n* = 230) (0.501–0.763)	0.689 (*n* = 118) (0.568–0.810)	0.713 (*n* = 118) (0.593–0.833)	N/A	N/A
B Groups	0.564 (*n* = 73) (0.424–0.703)	0.575 (*n* = 73) (0.436–0.714)	0.539 (*n* = 112) (0.429–0.649)	0.580 (*n* = 112) (0.472–0.689)	N/A	N/A

AROC: area under receiver operating characteristic curve; CI: confidence interval; BMI: body mass index; WC: Waist circumference; HDL-C: high-density lipoprotein cholesterol ratio; TG: triglyceride; A Groups: <90 cm for men, <80 cm for women; B Groups: ≥90 cm for men, ≥80 cm for women.

**Table 3:** Area under receiver operating characteristic curves for potential markers of insulin resistance.

Predicting variables	Male (AROC, 95% CI)	Female (AROC, 95% CI)
Clinical variables		
WC (cm)	0.793 (0.726–0.861)	0.766 (0.700–0.831)
HC (cm)	0.726 (0.649–0.804)	0.714 (0.641–0.787)
BMI (kg/m^2^)	0.746 (0.671–0.821)	0.772 (0.707–0.837)
Waist/hip	0.752 (0.678–0.827)	0.723 (0.655–0.791)
Biological variables		
TC (mmol/L)	0.489 (0.403–0.575)	0.411 (0.324–0.498)
HDL-C (mmol/L)	0.333 (0.252–0.414)	0.305 (0.229–0.382)
LDL-C (mmol/L)	0.553 (0.471–0.635)	0.534 (0.447–0.621)
TC/HDL-C	0.651 (0.569–0.734)	0.623 (0.545–0.702)
hsCRP (mg/L)	0.601 (0.514–0.689)	0.688 (0.612–0.764)

BMI: body mass index; WC: waist circumference; HC: hip circumference; TC: total cholesterol; LDL-C: low-density lipoprotein cholesterol; HDL-C: high-density lipoprotein cholesterol ratio; hsCRP: high sensitivity creactive protein; AROC: area under receiver operating characteristic curves; CI: confidence interval.
